# Stealing from the Heart: A Rare Case of Chest Pain Post-Coronary Artery Bypass Grafting

**DOI:** 10.14797/mdcvj.1239

**Published:** 2023-08-09

**Authors:** Prasanti A. Kotta, Joshua R. Hirsch, Umair Khalid, Ali E. Denktas

**Affiliations:** 1 Baylor College of Medicine, Houston, Texas, US

**Keywords:** coronary subclavian steal, subclavian artery stenosis, computed tomography angiogram, subclavian artery angiogram, bilateral blood pressure differential

## Abstract

A 70-year-old veteran with prior triple vessel coronary artery bypass grafting (CABG) presented with exertional chest pain. His work-up revealed > 40 mm Hg bilateral upper extremity blood pressure difference. Chest computed tomography and invasive angiography revealed severe stenosis at the ostium of the left subclavian artery, proximal to the origin of the left internal mammary artery to left anterior descending artery graft (LIMA-LAD). A diagnosis of coronary subclavian steal syndrome (CSSS) was made, and carotid-subclavian bypass was performed. This case outlines when to suspect CSSS, an approach to its diagnosis, and the importance of its timely management.

## Case Presentation

A 70-year-old veteran presented to his local Veterans Affairs Hospital with exertional chest pain. He described central, heavy chest pain and dizziness with exertion that worsened when using his left upper extremity. His medical history was significant for coronary artery disease, triple vessel coronary artery bypass grafting (CABG) with a left internal mammary artery to left anterior descending artery graft (LIMA-LAD), saphenous vein graft (SVG) to obtuse marginal, and SVG to right coronary artery 15 years ago. He also had atrial fibrillation, insulin-dependent diabetes, hypertension, and hyperlipidemia. Cardiovascular examination revealed a regular pulse, normal S1 and S2 with no murmurs, gallops, or rubs, nondisplaced point of maximal impulse in the fifth intercostal space at the midclavicular line, and symmetric palpable pulses in the upper and lower extremities bilaterally. He was hemodynamically stable, with a blood pressure of 160/85 in right arm, blood pressure of 125/85 in left arm, and oxygen saturation of 98% on room air. Sitting, lying, and standing blood pressures showed no orthostatic blood pressure changes.

**Figure 1:**
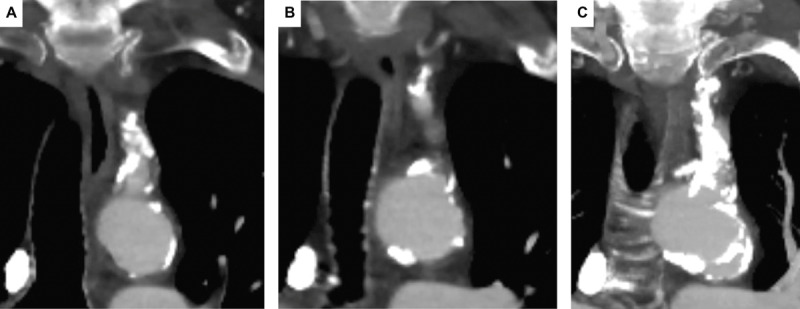
Computed tomography angiogram of the chest with visualization of the aortic arch and its branches, specifically **(A)** the proximal aspect of left subclavian artery with calcific stenosis, **(B)** the mid portion of left subclavian artery close to origin of vertebral artery, and **(C)** maximal intensity projection image showing distribution of calcification in the left subclavian artery.

**Figure 2:**
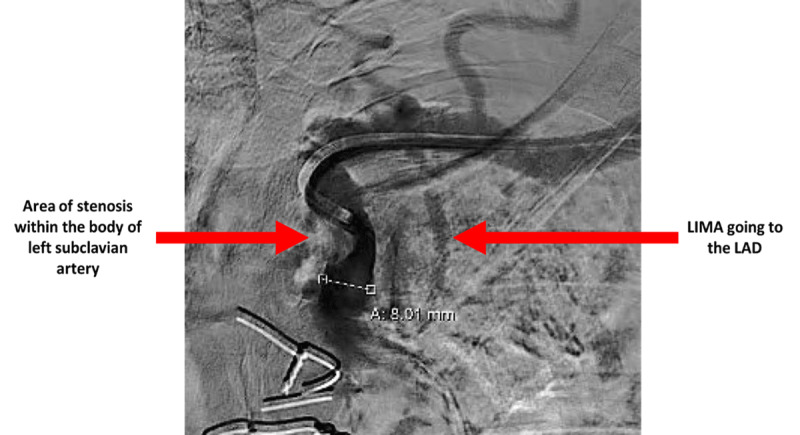
Invasive subclavian artery angiogram with red arrows pointing to severe proximal subclavian artery stenosis.

**Figure 3:**
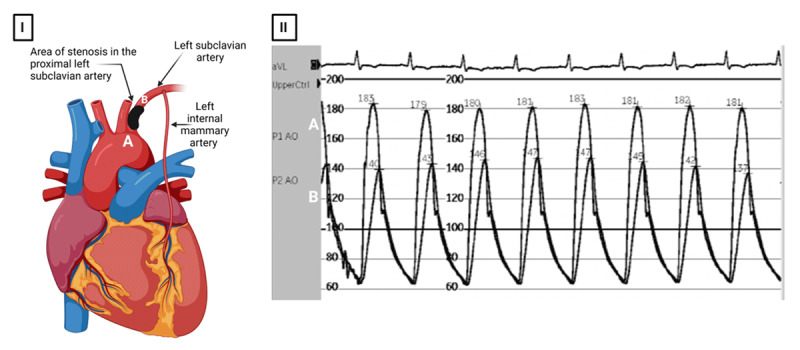
(I; left panel) Simultaneous pressure tracings taken proximal **(A)** and distal **(B)** to the stenosis through simultaneous femoral access and left radial access, respectively. (II; right panel) Pressure tracings demonstrate a peak-to-peak gradient ~ 40 mm Hg across the subclavian artery stenosis (> 20 mm Hg is considered hemodynamically significant). Created with BioRender.com.

Cardiac biomarkers, including serial troponin and brain natriuretic peptide, were within normal limits. A 12-lead electrocardiogram demonstrated sinus rhythm with no acute ischemic changes. A transthoracic echocardiogram demonstrated mildly depressed left ventricular ejection fraction of 40% to 45% with no regional wall motion abnormalities or valvular defects. A computed tomography (CT) angiogram of the chest, performed to further investigate the bilateral blood pressure differential, demonstrated severe stenosis at the ostium of the left subclavian artery proximal to the origin of the LIMA and a patent LIMA-LAD graft (Figure 1, Video 1). Given the patient’s high pretest probability and concerning symptoms, noninvasive ischemic evaluation was deferred and a direct coronary and subclavian artery invasive angiogram was subsequently performed. It demonstrated significant proximal subclavian artery stenosis (Figure 2, Video 2). Simultaneous pressure tracings were measured, obtained through simultaneous femoral and radial access into the ascending aorta and left subclavian artery respectively, proximal and distal to the subclavian artery stenosis (Figure 3 I). The pressure tracings revealed a peak-to-peak pressure gradient of approximately 40 mm Hg across the area of subclavian stenosis (Figure 3 II).

**Video 1:** A computed tomography angiogram of the chest showing severe stenosis at the ostium of the left subclavian artery proximal to the origin of the LIMA and a patent LIMA-LAD graft; see also at https://youtube.com/shorts/i4G9p7HvCcY. LIMA: left internal mammary artery; LIMA-LAD: LIMA to left anterior descending artery

**Video 2:** A direct coronary and subclavian artery invasive angiogram showing significant proximal subclavian artery stenosis; see also at https://youtu.be/2FcTAytfCdg.

**Figure 4:**
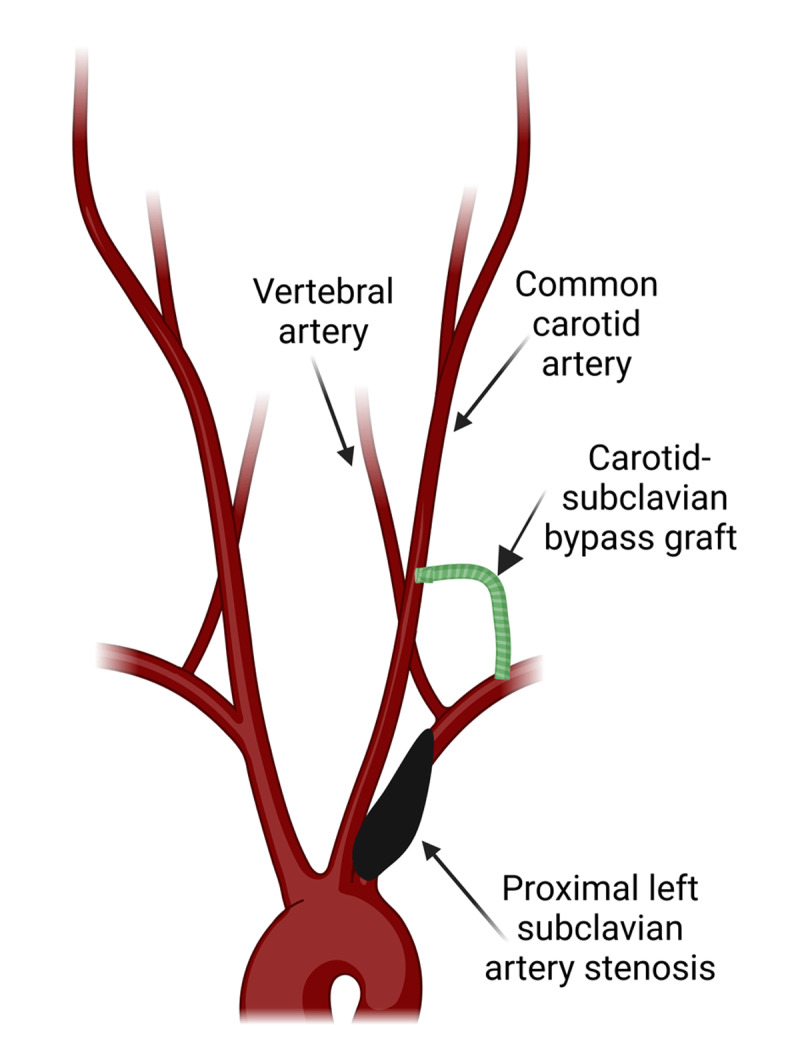
Graphic illustrates a carotid-subclavian bypass graft. Created with BioRender.com.

The large stenosis identified was not amenable to percutaneous intervention, with the area of stenosis too close to the origin of the ipsilateral vertebral artery, risking blockage of the vertebral artery with stent placement. The patient was referred for surgical evaluation and management. While awaiting vascular surgery, care was taken to ensure blood pressure was measured and treatment titrated to the side distal to the stenosis since blood pressures are the lowest here. He underwent a carotid-subclavian bypass using an 8-mm Dacron graft (Figure 4) to restore flow to the subclavian artery distal to the stenosis. Following surgery, the patient noted improvement in anginal symptoms, and the blood pressure differential between the two upper extremities corrected to < 15 mm Hg. He was discharged home with improvement in his functional status. He remained symptom free at 3- and 6-month follow-up.

## Discussion

This case illustrates coronary subclavian steal syndrome (CSSS), a phenomenon that occurs in patients with a LIMA-LAD graft and coexisting hemodynamically significant proximal subclavian artery stenosis (SAS). The SAS limits blood flow into the distal left subclavian artery, including the LIMA-LAD graft, restricting coronary blood flow to the territory being supplied by the LAD. During stress, a supply-demand mismatch causes anginal symptoms and, if persistent, can lead to ischemia, infarction, and sudden cardiac death.^1,2^

In this case, our patient experienced angina precipitated particularly by left upper extremity exertion, where demand-driven vasodilation of left upper extremity arteries leads to increased steal from the LIMA-LAD graft, exacerbating the reduced blood flow into the anterior myocardium. In patients whose SAS is worse than the native LAD stenosis, retrograde blood flow from the native LAD through the LIMA graft into the subclavian artery also occurs, leading to “coronary-subclavian steal.”^3^ CSSS can quickly transform from a stable presentation to rapidly progressive myocardial ischemia, hence timely and aggressive management is needed to prevent these complications.

CSSS remains an uncommon complication; however, prevalence is increasing with greater CABG procedural frequency and life expectancy after CABG. SAS is seen in up to 12% of patients with CABG and known peripheral arterial disease.^2^ CSSS affects 0.2% to 6.8% of CABG patients with a LIMA-LAD graft; the mean time between CABG surgery and development of CSSS is about 9.0 ± 8.4 years.^4^ However, the diagnosis of CSSS remains challenging, with low levels of suspicion among clinicians and current prevalence likely being underestimated due to underdiagnosis. A thorough chest pain history with clarification of exacerbating features including triggers, laterality of exacerbating features, and associated symptoms can be helpful in raising suspicion for the diagnosis.

Current guidelines lack recommendations in screening for SAS.^5^ A clinically significant SAS produces a difference in systolic blood pressure between the right and left upper extremity of 15 mm Hg to 20 mm Hg or more. A difference in systolic blood pressure > 15 mm Hg between two arms can be the only initial manifestation in the absence of symptoms. Thus, measurement of bilateral brachial blood pressures during routine annual visits after CABG can be used to screen for the interval development of SAS. In those with a blood pressure differential > 15 mm Hg, CT or magnetic resonance angiography (MRA) should be considered to evaluate for SAS. There are also case reports of pre-existing SAS before CABG and LAD-LIMA grafting compromising graft function, leading to cardiogenic shock post-CABG, suggesting possible benefit for routine subclavian artery stenosis screening using invasive or CT coronary angiography during pre-CABG evaluation.^6,7^

The 2011 European Society of Cardiology guidelines recommend percutaneous treatment as the first-line treatment for symptomatic CSSS.^8^ However, certain anatomical factors, including proximity to the ipsilateral vertebral artery, might preclude this in favor of surgical intervention via carotid-subclavian bypass, as in our case.^9,10^ Multimodality imaging, including CT or MRA, and duplex ultrasound can help quantify the SAS, distinguish flow characteristics, and visualize the relationship of the subclavian artery to the surrounding vascular structures, including the vertebral artery and LIMA graft.

Another important lesson from this case is that blood pressure treatment should be titrated to blood pressure measurements on the side distal to the stenosis, given that blood pressures are the lowest here. Titrating blood pressure treatment to the contralateral upper extremity can result in low blood pressures distal to the SAS, further reducing blood flow into the LIMA-LAD graft. Permissive hypertension on the contralateral upper extremity may be required to ensure adequate blood flow into the CABG graft distal to the SAS.

## Conclusion

The workup of chest pain in patients who have undergone CABG can be significantly challenging. Beyond more common intracoronary stenoses, extracoronary and extra-graft lesions, such as SAS, also may be implicated in compromised coronary blood flow. CSSS is a manifestation of SAS rarely found in patients who have undergone CABG. The prevalence of CSSS is increasing with greater CABG procedural frequency and life expectancy after CABG. The diagnosis of CSSS remains challenging, and a high degree of clinical suspicion is warranted to identify this phenomenon. A thorough chest pain history with clarification of exacerbating features including triggers, laterality of exacerbating features, and associated symptoms is helpful. Bilateral upper extremity blood pressure measurements during routine annual visits after CABG can be used to screen for the interval development of SAS. When undiagnosed and left untreated, CSSS can lead to angina, acute coronary syndrome, and sudden cardiac death. Permissive hypertension on the contralateral upper extremity may be required to ensure adequate blood flow into the CABG graft distal to the SAS.
